# Ultrahigh evaporative heat transfer measured locally in submicron water films

**DOI:** 10.1038/s41598-022-26182-2

**Published:** 2022-12-26

**Authors:** Xiaoman Wang, S. Arman Ghaffarizadeh, Xiao He, Alan J. H. McGaughey, Jonathan A. Malen

**Affiliations:** Department of Mechanical Engineering, Carnegie Mellon University, Pittsburgh, PA 15213 USA

**Keywords:** Mechanical engineering, Nanoscience and technology

## Abstract

Thin film evaporation is a widely-used thermal management solution for micro/nano-devices with high energy densities. Local measurements of the evaporation rate at a liquid-vapor interface, however, are limited. We present a continuous profile of the evaporation heat transfer coefficient ($h_{\text {evap}}$) in the submicron thin film region of a water meniscus obtained through local measurements interpreted by a machine learned surrogate of the physical system. Frequency domain thermoreflectance (FDTR), a non-contact laser-based method with micrometer lateral resolution, is used to induce and measure the meniscus evaporation. A neural network is then trained using finite element simulations to extract the $h_{\text {evap}}$ profile from the FDTR data. For a substrate superheat of 20 K, the maximum $h_{\text {evap}}$ is $1.0_{-0.3}^{+0.5}$ MW/$\text {m}^2$-K at a film thickness of $15_{-3}^{+29}$ nm. This ultrahigh $h_{\text {evap}}$ value is two orders of magnitude larger than the heat transfer coefficient for single-phase forced convection or evaporation from a bulk liquid. Under the assumption of constant wall temperature, our profiles of $h_{\text {evap}}$ and meniscus thickness suggest that 62% of the heat transfer comes from the region lying 0.1–1 μm from the meniscus edge, whereas just 29% comes from the next 100 μm.

## Introduction

Spatial resolution of amplified evaporation rates in nanometer- and micrometer-thick liquid films, as are found in menisci, is a long-standing challenge^1–4^. Accurate measurements require submicron lateral precision and a modeling framework to interpret the results. Experimental measurements have probed evaporation in the macroscopic extended meniscus, where the evaporation heat transfer coefficient takes on its bulk value of 0.001–0.1 MW/$\text {m}^2$-K^5–7^. Theory intriguingly suggests an up to three orders-of-magnitude enhancement of the evaporation rate, and hence the heat transfer rate, in the thin film region of the meniscus, but these predictions have not yet been validated^8–13^.

**Figure 1:**
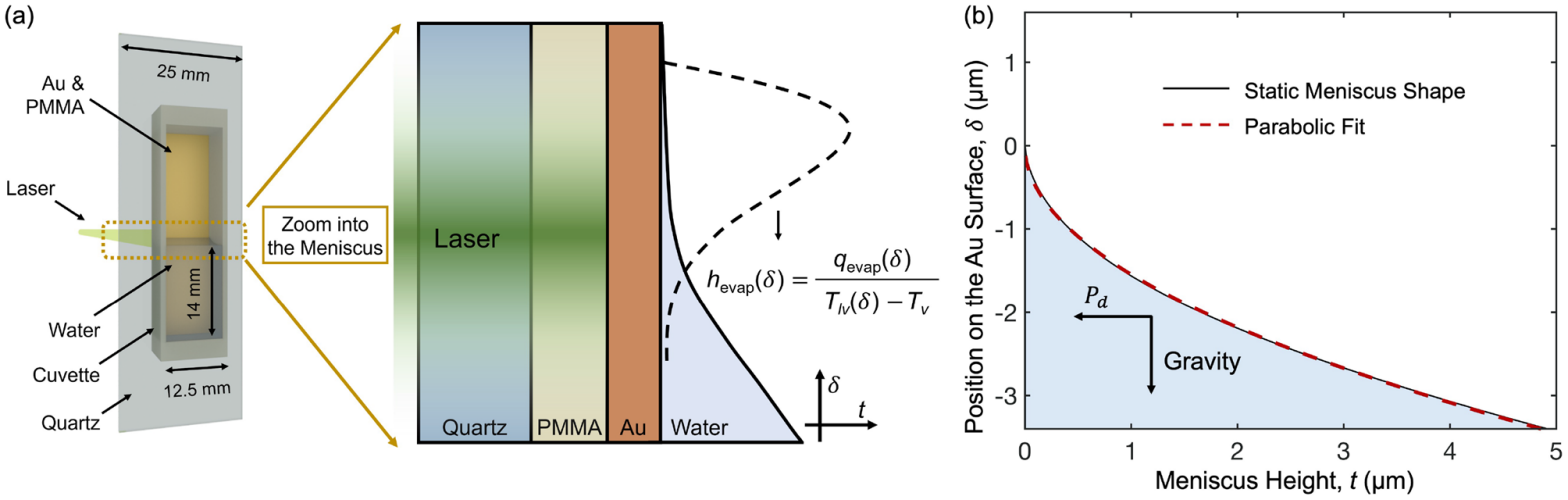
(**a**) Rendering of the experimental setup. The pump laser is incident from the back of the quartz slide and transmits through the PMMA layer to periodically heat the gold layer. The laser power has a Gaussian profile in the radial direction. The dashed line in the right panel illustrates the expected shape of the $h_{\text {evap}}$ profile^32,39^. (**b**) Static meniscus shape calculated from the augmented Young-Laplace equation without retardation effects. The red dashed curve is a parabolic fit.

The evaporation rate from a thin liquid film is controlled by a competition between the film thermal resistance and a suppressed liquid pressure. The latter results from the disjoining pressure $P_d$, which measures the interaction strength between the solid substrate and the liquid film. A smaller film thickness: (i) Decreases the thermal resistance, leading to a higher superheat at the liquid-vapor interface, which enhances evaporation, and (ii) Increases $P_d$, which suppresses evaporation^8–10,14^. These competing effects result in a non-monotonic profile for the evaporation heat transfer rate, as shown schematically in Fig. 1a. Quantifying this profile will reveal pathways to amplify heat transfer in micro/nanostructure thermal solutions used to manage high power density electronics, where single-phase air/liquid cooling cannot match the demand^15–21^. The efficiency of solar thermal generation^22–24^ and desalination processes^25,26^ will also be improved by engineering evaporation in thin liquid films to obtain high mass fluxes.

Experimental thin liquid film evaporation studies are often conducted by extracting the temperature profile along a meniscus on a heated surface. Infrared cameras^11–13^ and thermocouples^8–10,27^ with spatial resolution from 6 μm to 2 mm have been used to measure local temperatures. Reported heat flux and/or temperature profiles demonstrate enhanced heat transfer near the edge of the meniscus (i.e., the three-phase contact line). As an alternative, Höhmann et al.^28^ used thermochromic liquid crystals (TLCs) with a 1 μm spatial resolution. TLCs, however, suffer from limited life-spans and high measurement uncertainty^12,29^. Non-contact laser-based methods have also been used to study liquid-vapor phase change. Park et al. used ultrafast pump-probe spectroscopy to study the evaporation of a thin liquid film. They obtained the time-dependent film thickness response to a picosecond pump optical pulse, but did not report an evaporation rate profile^30^. Time-domain thermoreflectance was used by Mehrvand and Putnam to study microlayer evaporation in single bubbles during flow boiling of water.^4^ More recently, Che et al. combined time-domain thermoreflectance and numerical analysis to study the evaporation of an octane liquid film^31^. They report the variation of the overall heat transfer coefficient along the meniscus, obtaining a maximum value of 0.44 MW/$\text {m}^2$-K. This value includes the conductive thermal resistance of the liquid. Because Che et al. average over a 10 μm laser spot diameter, their overall heat transfer coefficient profile cannot resolve values that are less than 2 μm from the meniscus edge. Despite these advances, isolation of the evaporative heat transfer coefficient with microscale resolution across the entire meniscus has not been obtained experimentally.

The objective of this work is to obtain a continuous profile for the evaporation heat transfer coefficient, $h_\text {evap}$, for a thin water film using experimental measurements interpreted by a machine learned surrogate of the physical system. As shown in Fig. 1a, $h_{\text {evap}}$ at a position $\delta$ measured from the start of the meniscus is defined as the ratio of the evaporation heat flux $q_{\text {evap}}$ to the temperature difference between the interface and the vapor ($T_{lv} - T_v$)^2,32^. Water enclosed in a vertically-mounted cuvette forms a meniscus on its walls. At an ambient temperature of 295 K, the experiments are conducted using frequency domain thermoreflectance (FDTR), a non-contact laser-based method. A data-driven framework that uses finite element simulations to train a neural network is developed to extract the $h_\text {evap}$ and meniscus thickness profiles from the experimental results. At a substrate temperature increase of 20 K, $h_{\text {evap}}$ peaks at $1.0_{-0.3}^{+0.5}$ MW/$\text {m}^2$-K at a location $89_{-38}^{+88}$ nm from the edge of the meniscus, where the film thickness is $15_{-3}^{+29}$ nm. The evaporation heat transfer coefficient decreases to the set bulk value of 0.01 MW/$\text {m}^2$-K at a location $0.92_{-0.56}^{+0.56}$ μm from the edge of the meniscus, where the film thickness is $1.6_{-1.4}^{+1.8}$ μm. The peak value is two orders of magnitude larger than the heat transfer coefficient for single-phase liquid forced convection^7^.

## Experimental setup

FDTR is an experimental approach primarily used to measure the thermal conductivity of solid thin films and the thermal conductances of the interfaces between solids^33–35^. The measurement is achieved by analyzing the phase lag between the periodic heat flux deposited by a periodically modulated pump laser and the induced periodic surface temperature oscillation measured by a coaxial probe laser. This phase lag is typically fit to an analytical solution to the heat diffusion equation^36^, from which the unknown properties are extracted. See “Materials and methods” for FDTR details.

We apply FDTR to an evaporating meniscus to extract $h_{\text {evap}}$ and thickness profiles. The schematic in Fig. 1a illustrates the setup of a quartz slide with a 200 nm spin coated polymethyl methacrylate (PMMA) layer, onto which a 70 nm gold film is sputtered. Deionized water is the working fluid. To contain the liquid and form the meniscus, a cuvette is sealed onto the slide through a custom-built frame by applying mechanical pressure. The gold film serves as the transducer needed to absorb the pump laser and to provide a temperature dependent reflectance (i.e. thermoreflectance) for the FDTR measurements^33^. The two incoming lasers, which have an effective spot diameter of approximately 3.4 μm, transmit coaxially through the quartz lid from the back side and deposit heat into the gold film, which leads to evaporation of the water meniscus. The average power absorbed by the gold film is 600 μW and a superheat of around 20 K is achieved. The PMMA layer serves two purposes: (i) To channel heat to the meniscus by acting as a thermal insulation barrier on the quartz side, which enhances the sensitivity of the measured phase lag to $h_{\text {evap}}$, and (ii) To improve the adhesion of the gold to the quartz and prevent its delamination under external effects, such as environmental humidity and thermo-mechanical stresses^37,38^. See “Materials and methods” for fabrication details.

**Figure 2:**
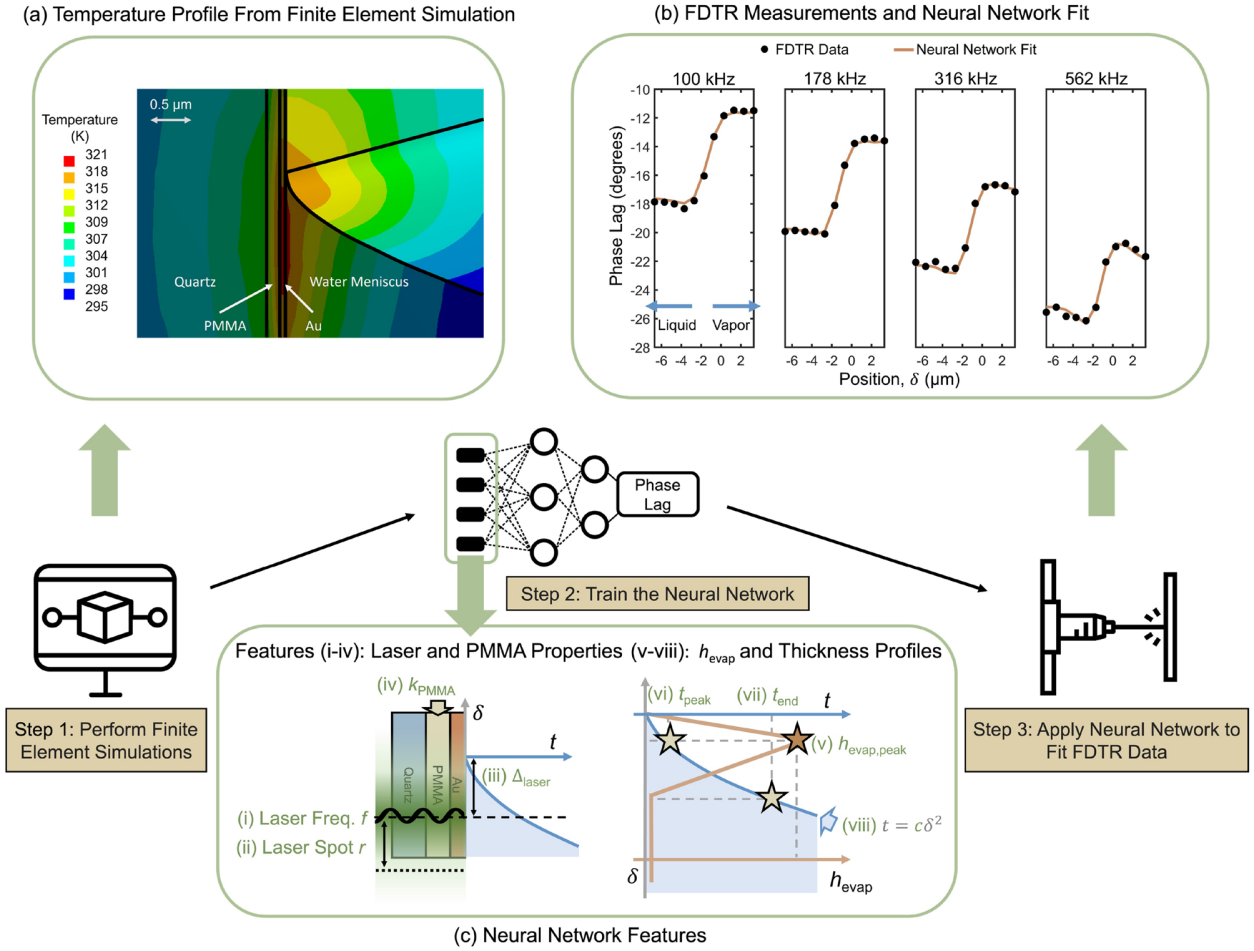
The three-step modeling framework includes finite element simulations, neural network training, and neural network fitting. (**a**) Finite element simulation of the temperature distribution in an FDTR experiment. The temperature increase is caused by a periodic heating and the phase lag is extracted from the spatially weighted gold temperature. (**b**) FDTR phase lag along the scanned region of the meniscus at four frequencies. The black circles are the measurements, obtained by scanning from the bulk liquid to the bulk vapor. The beige curves show the predictions from the trained neural network that minimize the mean square error. (**c**) The eight input features [(i)–(viii)] for the neural network.

The stage is moved vertically across the lasers in increments of ${1}$ μm, starting from the bulk liquid, across the meniscus, and into the bulk vapor. At a given position, four laser modulation frequencies (100 kHz, 178 kHz, 316 kHz and 562 kHz) are probed. The maximum frequency is set by the thermal penetration depth, which decreases as frequency increases [Eq. (S6)], making FDTR less sensitive to the liquid-vapor interface. The four subplots of Fig. 2b, each of which corresponds to one frequency, show the measured phase lag (plotted as black circles) as a function of position along the meniscus. While one scan is shown in Fig. 2b, six scans taken on different days are used in the analysis that follows. See “Materials and methods” for details on how the meniscus was located.

The standard analytical FDTR model assumes uniform layer thicknesses within its framework of the cylindrical heat diffusion equation^40,41^. As shown in Fig. 1b, a calculation based on the augmented Young–Laplace equation estimates a change in film thickness of up to 5 μm within the laser spot diameter of 3.4 μm. The uniform thickness FDTR model can therefore not be used to extract the $h_{\text {evap}}$ and film thickness profiles from the phase lag data. See Sect. S3 for information on the theoretical meniscus shape calculation.

## Data-driven modeling framework

We developed a data-driven modeling framework to extract the $h_{\text {evap}}$ and film thickness profiles from the FDTR measurements. The three-step workflow is shown in Fig. 2. In Step 1, finite element simulations representative of the experimental setup are performed. In Step 2, the known inputs (including parameters related to the laser, meniscus, and materials) and output (the phase lag) from the finite element simulations are used to train a neural network. In Step 3, FDTR results are fitted to the trained neural network to obtain the optimized neural network inputs, which include the $h_{\text {evap}}$ and film thickness profiles.

A multilayer feedforward neural network, as an universal function approximator, provides a high degree of flexibility to investigate a rich input parameter space^42^. To construct the neural network, eight input features [Fig. 2c], were selected to describe the FDTR experiment. Four have small uncertainties: (i) the laser frequency *f*, (ii) the laser spot radius *r*, (iii) the laser position relative to the starting point of the meniscus $\Delta _\text {laser}$, and (iv) the thermal conductivity of the PMMA layer $k_\text {PMMA}$. The $h_{\text {evap}}$ profile is approximated as three straight lines with three features: (v) The peak value $h_{\text {evap,peak}}$, achieved at (vi) a film thickness of $t_{\text {peak}}$, and (vii) the film thickness $t_{\text {end}}$ where $h_{\text {evap}}$ reduces to the set bulk value of 0.01 MW/$\text {m}^2$-K^43–45^. The meniscus thickness profile is described as $t(\delta )=c\delta ^2$, where (viii) *c* is the interface shape coefficient. When the predicted static meniscus profile is fitted to this functional form, a correlation coefficient of 0.9997 is achieved with $c=0.42$ μ$\mathrm{m}^{-1}$, as shown in Fig. 1b. The value of *c* is expected to increase when the meniscus is heated^32^.

Finite element simulations that mimic the FDTR experiments, based in the transient mechanical module in ANSYS Workbench^46^, are used to train the neural network. The pump laser is modeled as a periodic heat flux at the gold/PMMA interface at a specified frequency with a Gaussian radial profile. The temperatures of the nodes at the gold/PMMA interface are extracted and weighted spatially by the probe laser profile to calculate the average temperature at any instant in time. The phase lag is calculated as the phase difference of the applied heat flux and this average temperature. This approach is validated by comparing the calculated phase lag against the analytical prediction in bulk liquid and bulk vapor systems. See “Materials and methods” and Fig. S5 for details on the finite element simulations and their validation. Evaporation from the meniscus is modelled as a convection boundary condition at the liquid-vapor interface specified by the $h_{\text {evap}}$ profile. Fluid dynamics within the meniscus are not considered. Prior studies have shown that the effects of inertial forces^47^, thermocapillary convection^48^, and thermal resistance at the interface^49,50^ on the fluid flow and heat transfer in the evaporating thin film are small. As we will show, our model’s predictions are in good agreement with the experimental results, indicating that fluid dynamics can be neglected in this analysis.

By varying the inputs (i)-(viii) over a rich parameter space, 2653 distinct evaporating meniscus systems are simulated by the finite element simulations (Step 1 in Fig. 2). The parameter space construction is discussed in Sect. S4. The finite element simulation results are used to train the neural network (Step 2 in Fig. 2). A three-layer neural network with hidden layer sizes of 12 and 15 is selected through the random search technique. See “Materials and methods” for details about the neural network structure. The correlation coefficient for the neural network prediction of the validation data is $0.9990\pm 0.0001$.

Once trained, the neural network becomes a surrogate for the finite element simulation and is used to fit the FDTR experimental data (Step 3 in Fig. 2). For each of the six data sets, the phase lags at the four frequencies are fit simultaneously. The Powell optimization technique is used to extract the feature values that minimize the mean square error (MSE) between the neural network-predicted and FDTR-measured phase lags. During the optimization process, frequency is fixed as a known parameter taken directly from the experiment. $k_{\text {PMMA}}$ is set to the literature value of $0.240\pm 0.005$ W/m-K^51^ with its uncertainty incorporated in the analysis by a uniform grid search over the reported range. Six features (*r*, $\Delta _\text {laser}$, $h_\text {evap,peak}$, $t_\text {peak}$, $t_\text {end}$ and *c*) are thus extracted from the Powell optimization. More information on the Powell optimization is provided in Sect. S6.

## Results and discussion

An example of the neural network phase lag prediction from Step 3 for one of the data sets is plotted in Fig. 2b as solid beige lines. The neural network predictions capture the FDTR data well at all frequencies and scan positions.

Neural networks of the same structure but implemented with different algorithmic factors yield different predictions^52,53^. Here, the effect of uncertainty in algorithmic factors is evaluated by changing the random seeds for: (i) Splitting the dataset into training and testing subsets,^52^ and (ii) Initializing the weights^53^. One thousand neural networks with different seeds are created. Each of the one thousand neural networks is then used to fit each of the six FDTR datasets through the Powell optimization. The set of six features resulting in the lowest MSE is extracted for each data set/neural network pair (i.e., we obtain six thousand values for the six features).

**Figure 3:**
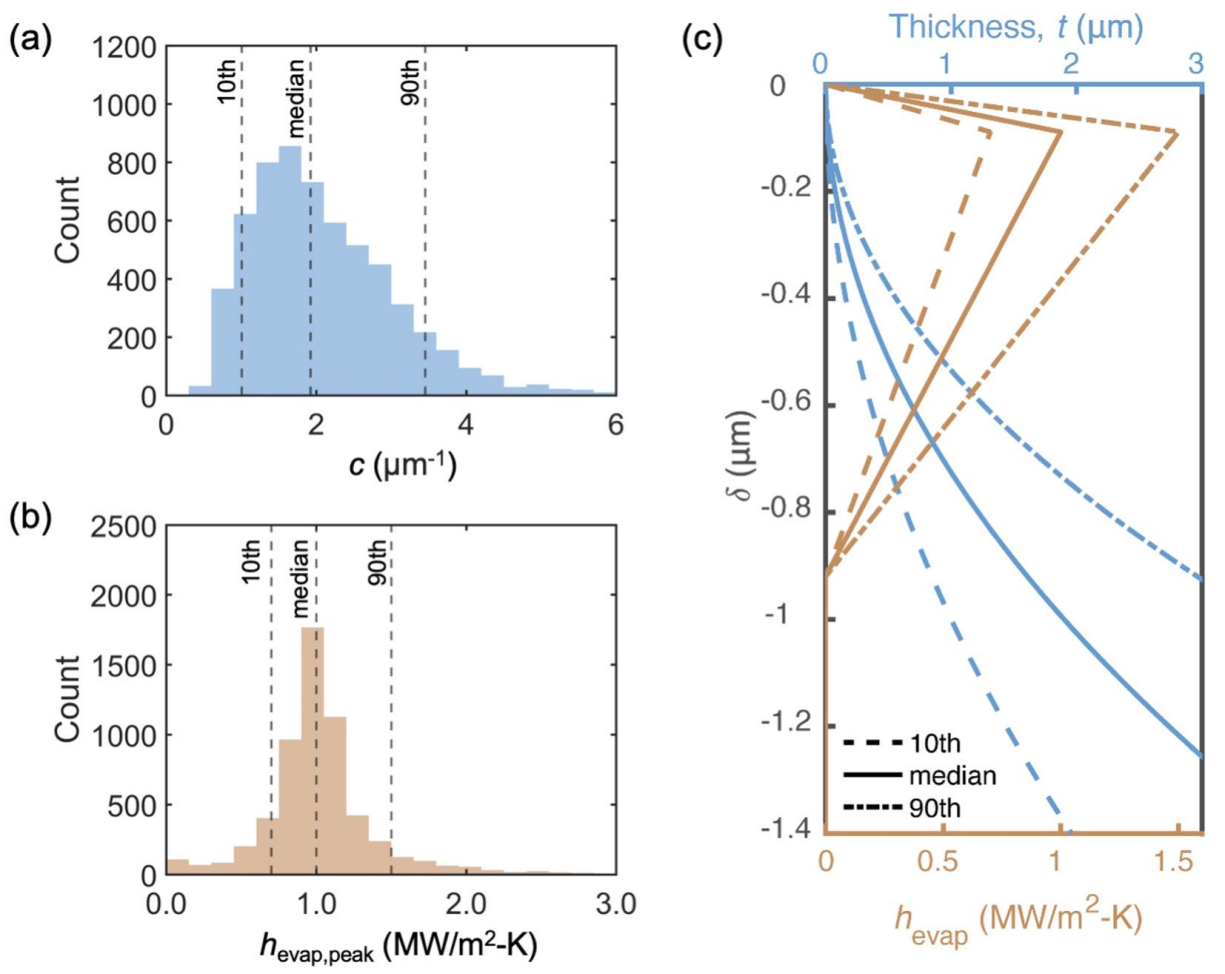
Distributions of (**a**) interface shape coefficient *c* and (**b**) $h_{\text {evap,peak}}$ from the neural network run with randomized seeds. The dashed vertical lines show the 10th percentile, median value, and 90th percentile. (**c**) $h_{\text {evap}}$ and meniscus shape profiles. The solid lines indicates the profiles using the median values. The dashed lines and dashed dotted lines indicate the profiles using the 10th and 90th percentile values.

Of the extracted features describing the shape of the meniscus and the continuous $h_\text {evap}$ profile [features (v)–(viii) in Fig. 2c], the neural network is most sensitive to $h_{\text {evap,peak}}$ and the coefficient *c*, as shown in Fig. S8b. To analyze the variation in the obtained values, we plotted histograms of $h_{\text {evap,peak}}$ and *c* for each of the six data sets (Fig. S9) and investigated their similarities by the two-sample Kolmogorov-Smirnov null hypothesis test (Sect. S8). The results do not prove that the distributions are drawn from the same population. Potential sources of the differences are variations in the lab environment and scan positions because the six FDTR data sets were taken on six different days. That being said, the median value of each histogram is within the 10th and 90th percentiles of the other histograms for both features. To illustrate the data spread, the six histograms are combined to obtain the distributions for *c* and $h_{\text {evap,peak}}$ plotted in Fig. 3a and b. The distributions are non-Gaussian. In subsequent discussion, the 10th and 90th percentile values are used to quantify the uncertainty. Using these statistics, the $h_{\text {evap}}$ and thickness profiles are plotted in Fig. 3c. For the $h_{\text {evap}}$ profiles, the median values of $t_{\text {peak}}$, $t_{\text {end}}$, and *c* are used.

Wang et al. theoretically studied changes in an octane meniscus shape for different substrate temperatures^32^. For a 20 K increase in the substrate temperature, they found the interface shape coefficient *c* to increase by a factor of eight compared to the static meniscus (i.e., no superheat). Here, *c* for the static meniscus is 0.42 μ$\text {m}^{-1}$ [Fig. 1b]. For the calculated superheat of 20 K, the *c* extracted from our modeling framework is $1.9_{-0.9}^{+1.6}$ μ$\text {m}^{-1}$, which is approximately four times larger than the static value.

As shown in Fig. 3c, at single nanometer film thicknesses, high $P_d$ suppresses the evaporation. As the thickness increases, $P_d$ decreases and the evaporation rate increases. $h_{\text {evap}}$ reaches its largest value of $1.0_{-0.3}^{+0.5}$ MW/$\text {m}^2$-K at $t_{\text {peak}}=15_{-3}^{+29}$ nm, which is $89_{-38}^{+88}$ nm from the meniscus edge. Further increasing the liquid film thickness increases the liquid conduction resistance. $h_{\text {evap}}$ thus decreases and reaches the set bulk value of 0.01 MW/$\text {m}^2$-K at $t_{\text {end}}=1.6_{-1.4}^{+1.8}$ μm, which is $0.92_{-0.56}^{+0.56}$ μm from the meniscus edge. Reported values from theoretical calculations of $h_{\text {evap,peak}}$ are between 0.8 and 8 MW/$\text {m}^2$-K for superheats between 3 and 50 K^39,54–56^. We note that the heating in an FDTR experiment is applied locally with a laser, resulting in a temperature distribution at the point of incidence, in contrast to the constant wall temperature in most past analyses.

We assess our results by performing two calculations of the mass flux ($\dot{m}$) profile. First, from the Hertz-Kundsen-Schrage (HKS) relation^1,14^,

1 $$\begin{aligned} \dot{m}''_{\text {evap,HKS}}=\frac{2\times \alpha (T_{lv})}{2-\alpha (T_{lv})}\sqrt{\frac{M}{2{\pi }R}}\left[ \frac{P_{eq}(T_{lv})}{\sqrt{T_{lv}}}-\frac{P_{v}}{\sqrt{T_{v}}}\right] , \end{aligned}$$

where $T_{lv}$ is the spatially-dependent liquid-vapor temperature, $\alpha$ is the mass accommodation coefficient at the liquid-vapor interface, *M* is the molar mass of the liquid, *R* is the universal gas constant, $P_v$ is the pressure in the vapor, and $P_{eq}$ is the so-called equilibrium pressure, which is the modified saturation vapor pressure under the influence of disjoining pressure and capillary pressure. Second, from a mass-energy balance as

2 $$\begin{aligned} \dot{m}''_{\text {evap,direct}} = \frac{h_\text {evap}(T_{lv})\times (T_{lv}-T_v)}{h_{fg}}, \end{aligned}$$

where $h_{fg}$ is the latent heat of vaporization. See Sect. S9 for details about the calculations from Eqs. (1) and (2).

We applied a temperature-dependent $\alpha$ profile, with $\alpha = 0.995$ at our peak mass flux, from the molecular dynamics simulations of Chandra and Keblinski^57^. The resulting peak value of $\dot{m}''_{\text {evap,HKS}}$ is 40% higher than the peak value of $\dot{m}''_{\text {evap,direct}}$, as shown in Fig. S10. Consistent with this result, Chandra and Keblinski found $\dot{m}''_{\text {evap,HKS}}$ to be 6-19% larger than that extracted directly from their self-consistent simulations^57^. They showed that their difference is due to a non-zero averaged vapor molecule velocity tangential to the interface, which violates a key assumption of the HKS relation. Given the spatial profile and lateral temperature gradients of the liquid-vapor interface, this assumption is likely violated in our experiments. An additional source of the discrepancy between our $\dot{m}''_{\text {evap,HKS}}$ and $\dot{m}''_{\text {evap,direct}}$ values is challenges in applying the HKS relation, especially the proper choice of $\alpha$^58^. For water, reported values span over four orders of magnitude, from $10^{-4}$ to 1^1,59^. A temperature-independent $\alpha$ of 0.8 makes the peak values of $\dot{m}''_{\text {evap,HKS}}$ and $\dot{m}''_{\text {evap,direct}}$ match.

**Figure 4:**
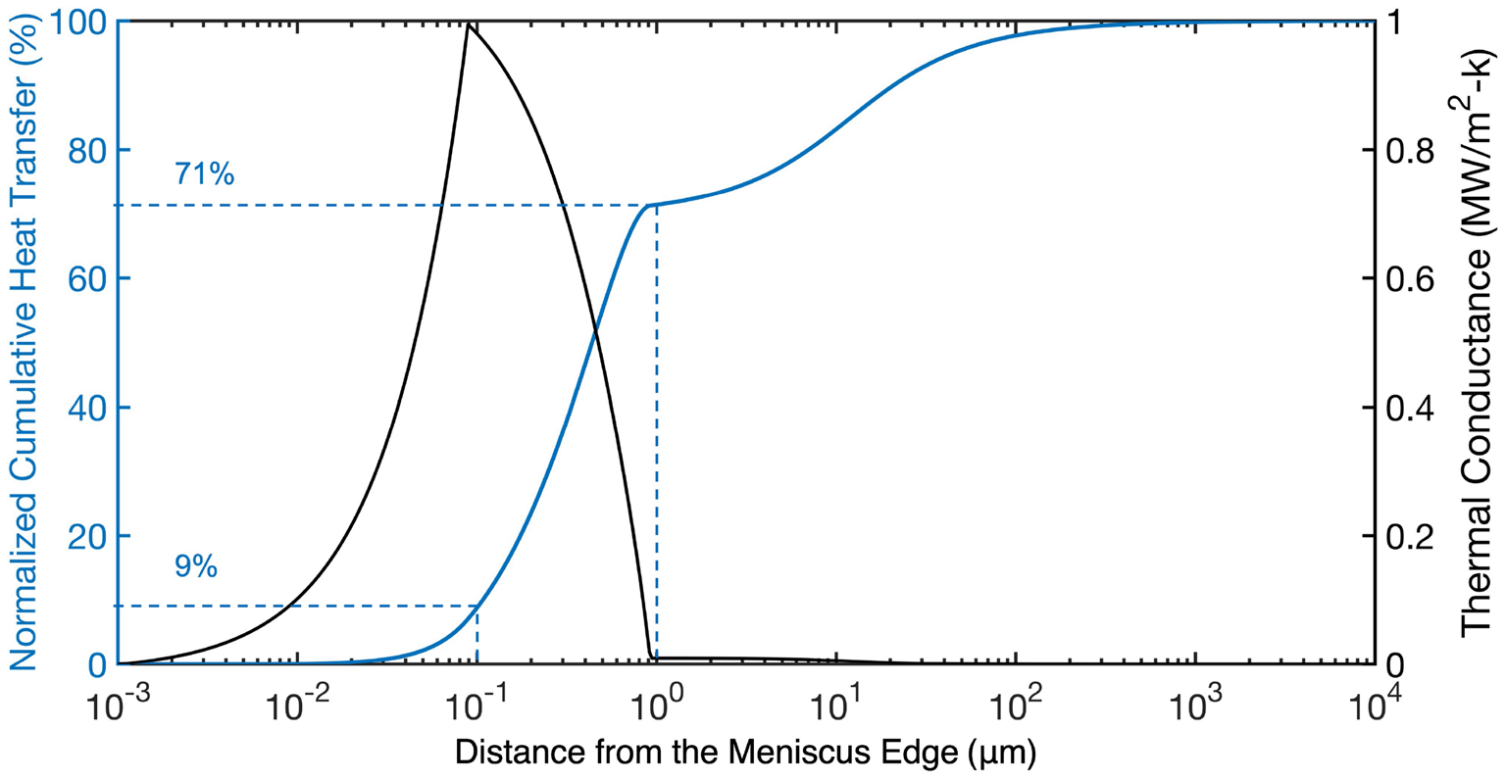
Thermal conductance profile across the meniscus (black line). The blue line shows the normalized cumulative heat transfer. The two vertical dashed lines mark the positions where the distances to the meniscus edge are 0.1 μm and 1 μm. The two horizontal dashed lines show the corresponding normalized cumulative heat transfer percentage, which are 9% and 71%.

Using the obtained $h_{\text {evap}}$ and thickness profiles, heat transfer in the meniscus under the assumption of a constant wall temperature can be studied. The spatially-dependent thermal conductance $G(\delta )$ between the wall and the vapor is calculated as

3 $$\begin{aligned} G(\delta ) = \frac{1}{1/h_{\text {evap}}(\delta )+t(\delta )/k_{\text {water}}}, \end{aligned}$$

where $k_{\text {water}}$ is the water thermal conductivity (0.6 W/m-K)^60^. The thermal conductance profile across the meniscus is shown as the black line in Fig. 4. The normalized cumulative heat transfer across the meniscus is calculated as the cumulative area under the thermal conductance profile, shown as the blue line in Fig. 4. 62% of the heat transfer comes from the region 0.1–1 μm from the meniscus edge, while only 29% comes from the next 100 μm.

## Conclusion

High spatial resolution thermoreflectance measurements and a finite element simulation/neural network interpretation of the results enabled us to obtain a continuous $h_{\text {evap}}$ profile in the thin film region of a water meniscus. The peak $h_{\text {evap}}$ is within the range of reported theoretical and experimental results and definitively quantifies competing effects from liquid conduction resistance and disjoining pressure. Under the assumption of constant wall temperature, our profiles of $h_{\text {evap}}$ and meniscus thickness define a target length scale of 0.1–1 μm for the design of surface features and pore sizes to enhance evaporation heat transfer rates. The framework we developed for analyzing FDTR data through training a neural network with representative computational results could be applied to interpret other thermal transport measurements that do not lend themselves to analytical solutions.

## Materials and methods

### Frequency domain thermoreflectance (FDTR)

In FDTR, the transducer layer (here, gold) is heated by a 488 nm continuous-wave pump laser (Coherent) that is intensity-modulated over a frequency range of 100-562 kHz. The periodic heating generates a periodic change in the surface temperature of the gold layer, with a phase lag relative to the heat flux that depends on the properties of the sample. Thermoreflectance of the gold layer causes modulation of a reflected 532 nm probe laser (Coherent). Its phase lag relative to the pump is monitored by a lock-in amplifier (Zurich Instruments Model HF2LI). A double-modulation scheme is implemented where a mechanical chopper is added in the probe path to remove coherent noise from the signal. The average power absorbed by the transducer layer is 600 μW, which was measured with a digital handheld optical power console (Thorlabs). From this value, the steady-state temperature rise of the gold is estimated to be 20 K with a periodic temperature amplitude that ranges from 6-13 K depending on the modulation frequency.

### Sample fabrication and setup

The water is confined by a demountable cuvette with a 5 mm light path purchased from Starna Cells, Inc. (Type 49). To ensure a clean environment for the evaporation experiment, the cuvette was cleaned with a nanostrip after purchase and filled with water in a class 100 cleanroom. The quartz substrate is from VWR Corp. (Grade GE124).

The PMMA layer is prepared by spin coating 495 PMMA onto the quartz slide, which is taped onto a 6 inch silicon wafer. The sample is first spun at 500 RPM for 6 s then accelerated to 4000 RPM for 60 s to achieve a thickness of $200\pm 4$ nm measured by profilometry. The coated slide is baked in vacuum at a temperature of 180 $^\circ$C for 120 s to cure. The transmittance of the PMMA-coated quartz slide is calculated at the FDTR wavelengths (pump: 488 nm, probe: 532 nm) using the Fresnel equation^61^. The reflection loss at the interface between air and the quartz slide is 4%, and that between the quartz and PMMA is below 1%, with negligible absorption in quartz and PMMA at the FDTR wavelengths^62,63^.

The gold layer is sputtered onto the fully-cured PMMA using the Perkin Elmer 6J Sputtering System. The thickness of the gold layer is measured to be $70\pm 1$ nm using profilometry. The typical RMS roughness of the sputtered gold from this system is 2 nm as measured by X-ray reflectivity. The electrical conductivity of the gold layer is measured using a four-point probe and used to calculate its thermal conductivity by the Wiedemann-Franz law.

Before assembling the sample, the quartz slide and cuvette are both cleaned with isopropyl alcohol and rinsed by deionized water in a class 100 cleanroom. To prevent any change in the water content, proper sealing between the cuvette and the quartz substrate is essential. To improve the seal, a high-temperature silicone gasket cut into the shape of the cuvette edge is placed between the cuvette wall and the quartz slide. The gasket is placed in compression by a 3D printed nylon 12 frame, as shown in Fig. S1. The setup is placed at room temperature for a 48-hour period before measurement to degas the water and ensure that there is no water level change. One measurement scan takes three to five hours.

### Meniscus location

During the FDTR experiment, the meniscus is located by moving the sample vertically downwards so that the fixed lasers scan from the bulk liquid to the bulk vapor, as shown in Fig. S2a. The total scan distance is 20 μm and the distance between each data point is 1 μm. In Fig. S2b, the abrupt change in signal locates the transition region.

### Finite element simulation

A temporally-periodic heat flux with a Gaussian profile in the radial direction is set to each node on the gold surface (the interface with PMMA) using the ANSYS Parametric Design Language (APDL) to simulate the FDTR pump laser. Because FDTR considers the phase lag between the heat flux and the surface temperature (gold surface), only the AC component of the laser heat flux is applied. After the simulation, the time series of temperatures for each node on the gold layer are output through APDL commands.

Evaporation is set as the convection boundary on the meniscus surface using the $h_{\text {evap}}$ profile. Even with the bulk value of 0.01 MW/m$^2$-K, the heat transfer by the phase change process is three orders of magnitude larger than that by natural convection^7^. Neglecting mass transfer and fluid dynamics simplifies the model, which enables exploration of fine increments in the parametric space. More information on the finite element simulation meniscus setup and validation is provided in Sect. S4.

### Neural network structure

A three-layer neural network with two hidden layers (12, 15) is selected through the random search technique using the Scikit-Learn package^64^. The logistic activation function has the best performance in terms of accuracy and training time. The limited-memory Broyden–Fletcher–Goldfarb–Shanno (L-BFGS) algorithm is used as the optimization solver. The max iteration is set to 10,000 and the tolerance is set to 0.001. More information on the random search is provided in Sect. S5.

## Supplementary Information

Supplementary Information.

## Data Availability

The datasets used and/or analysed during the current study available from the corresponding author on reasonable request.
