# Supplementary material for: Ultrahigh evaporative heat transfer measured locally in submicron water films
Source: Sci Rep. 2022 Dec 26;12:22353. doi: 10.1038/s41598-022-26182-2 (PMC9792458; doi:10.1038/s41598-022-26182-2)
Supplement: Supplementary file 1 — Supplementary Information. [file 41598_2022_26182_MOESM1_ESM.pdf]

Supplementary Material: Ultrahigh evaporative heat transfer measured locally in submicron water films

Xiaoman Wang[1,+], S. Arman Ghaffarizadeh[1,+], Xiao He[1], Alan J. H. McGaughey[1,*], and Jonathan A. Malen[1,*]

[1]Department of Mechanical Engineering, Carnegie Mellon University, Pittsburgh, Pennsylvania 15213, USA.

[*]mcgaughey@cmu.edu, jonmalen@andrew.cmu.edu

[+]these authors contributed equally to this work

S1 Experimental setup

The concentric pump and probe laser beams are moved vertically to scan a vertical meniscus enclosed in an optical cuvette. The cuvette is sealed by a quartz slide with gold sputtered in the middle section as the transducer layer necessary for the FDTR measurement. A layer of PMMA is sandwiched between the quartz slide and gold layer as thermal insulation and an adhesion layer. Sealing is achieved through mechanical pressure using a custom designed 3D-printed stage, as shown in Fig. S1. As can be seen from the rendering in Fig. S1(a), the 3D printed stage has two components: (i) a lid cover (shown in red) to apply mechanical pressure on the frame of the cuvette while leaving a slit open for the laser, and (ii) a mount (shown in grey) to hold the cuvette. A rubber gasket (shown in green) is cut into the shape of the cuvette edge to enhance the sealing. The assembly is shown in the rendering in Fig. S1(b). The photographs in Figs. S1(c) and S1(d) show the assembled stage and the lasers being shot onto the quartz slide.

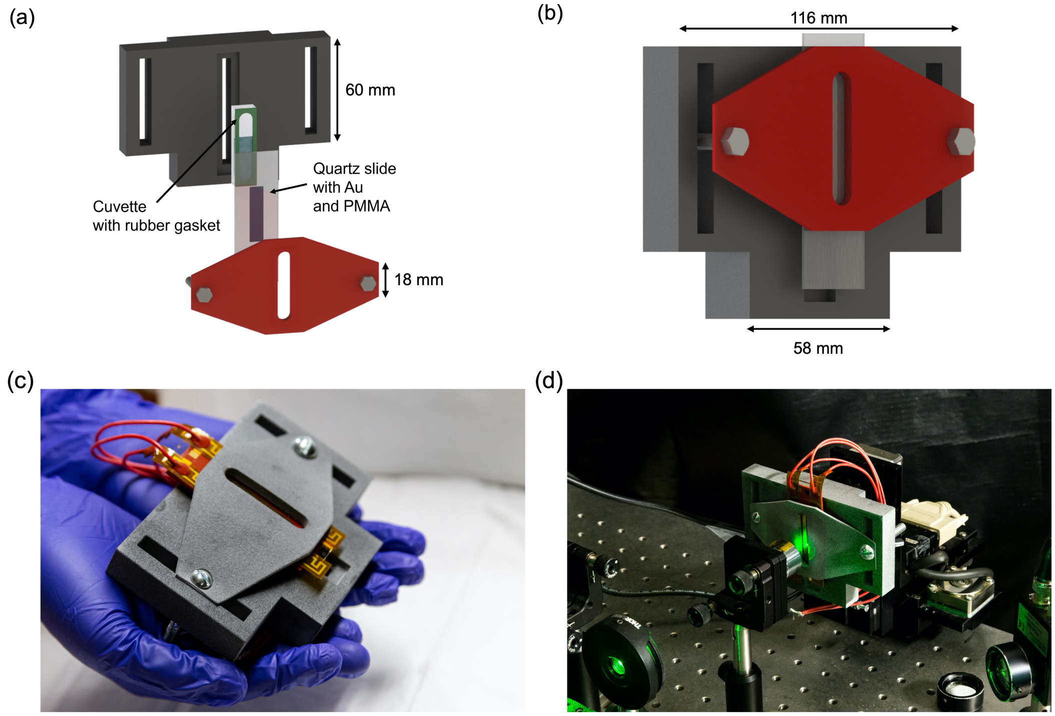

Figure S1. Renderings of (a) customized parts and (b) sample stage assembly. (c) Photograph of the sample stage. (d) Photograph of the laser beams shining onto the quartz slide. In (c) and (d), two resistance heaters are seen attached to the quartz slide for future temperature-controlled studies.

S2 Locate Meniscus

The sample is moved vertically downwards so that the fixed lasers scan from the bulk liquid to the bulk vapor region, as shown in Fig. S2(a). The experimental signal at a modulation frequency of 100 kHz is shown in Fig. S2(b), with the horizontal axis being the meniscus moving distance relative to the laser and the vertical axis being the phase lag between the pump and probe lasers. The total scan distance is 20 µm and the distance between each data point is 1 µm. A clear transition is evident in Fig. S2(b), which demonstrates where the meniscus region begins and ends.

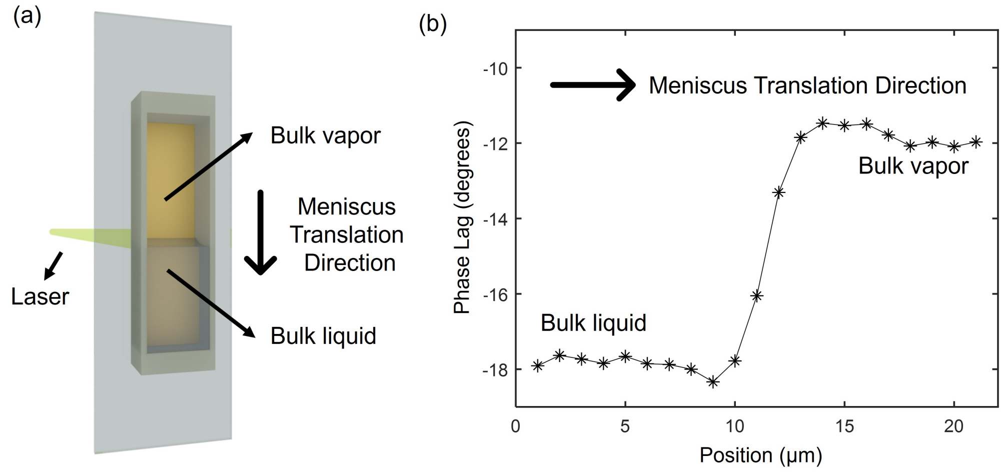

Figure S2. (a) Schematic drawing of lasers scanning along the meniscus. (b) Raw signal from FDTR at 100 kHz across the meniscus.

S3 Theoretical Meniscus Shape

FDTR has previously been used in systems where the layer thicknesses are uniform. To investigate the applicability of the associated analysis approach for a curved meniscus, its shape must be calculated by considering the forces near the water-Au contact. We follow the procedure described by Wang et al.[1] to determine the meniscus thickness δ as a function of position t measured from where it starts (Fig. S3).

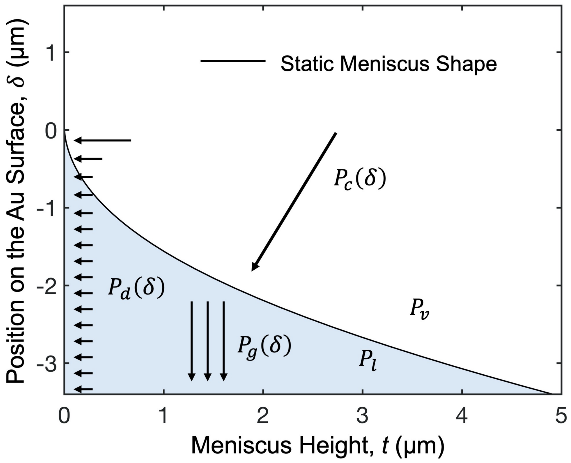

Figure S3. Force balance along the meniscus. P_d, P_c and P_g vary along the δ axis. Their directions are shown as black arrows.

The augmented Young-Laplace equation provides the force balance

$$P_v - P_l = P_c + P_d + P_g, \tag{S1}$$

where

$$P_c = \sigma \frac{t''}{(1+t'^2)^{2/3}} \equiv \sigma\kappa, \tag{S2}$$

$$P_d = \frac{A}{6\pi t^3}, \tag{S3}$$

$$P_g = \rho g(L-\delta). \tag{S4}$$

Here, P_v is the pressure in the vapor, P_l is the pressure in the liquid, P_c is the capillary pressure, P_d is the disjoining pressure, P_g is the pressure imposed by the gravity, σ is the water surface tension, κ is the curvature, A is the Hamaker constant, ρ is the liquid density, g is the acceleration due to gravity, and L

is the baseline position on the gold surface to evaluate the gravity, which disappears when derivatives are taken. By using the Hamaker constant, water is treated as a non-polar liquid. As such, the disjoining pressure only incorporates van der Waals interactions and does not include the retardation of the dispersion forces due to electrostatic interactions. The property values are provided in Table S1. The directions of the forces associated with P_d and P_g are shown in Fig. S3. Due to insufficient knowledge to the exact values for P_l, the meniscus shape is solved by taking the derivative of Eq. (S1) with respect to δ. With assumed negligible spatial variations in the liquid and vapor pressures, the meniscus shape can be obtained numerically for a non-evaporating and static meniscus.[1]

There are three boundary conditions. (i) The absorbed water film thickness at the start of the meniscus, $t(0)$, is calculated at a negligible wall superheat ($T_w - T_v$ = 0.005 K) from

$$t(0) = \left\{ \frac{A}{6\pi} \times \frac{1}{-\rho T_w R/M \ln\left[\sqrt{T_w/T_v} \times P_v/P_{sat}(T_w)\right] + P_v\sqrt{T_w/T_v} - P_{sat}(T_w)} \right\}^{\frac{1}{3}}, \tag{S5}$$

where M is the molar mass of water, R is the universal gas constant, T_w is the wall temperature, and P_{sat} is the saturation pressure. The calculated value is $t(0) = 3.3$ nm. (ii) The initial slope for a completely wetting film $t'(0) = 0$. (iii) The curvature in the far field κ_{far} is 350 m^{-1}, when δ_{far} is set as -5 μm and P_d is negligible. This curvature is calculated by solving Eq. (S1) with the cuvette depth of 5 mm and the contact angles (see Table S1) between the two solid bounds (the gold film and the cuvette window) without considering P_d, as shown in Fig. S4.

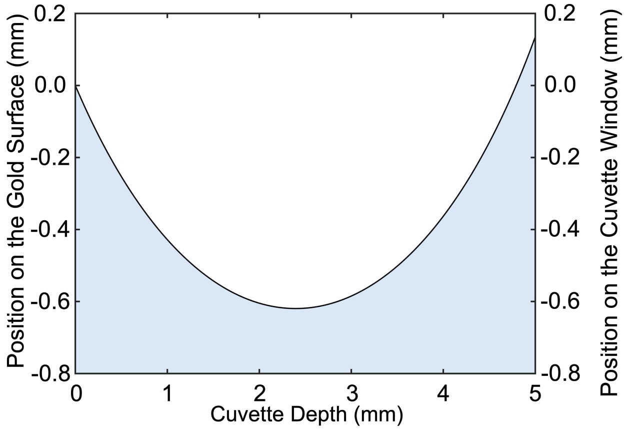

Figure S4. System used to calculate the curvature in the far field.

The theoretical calculation of the meniscus shape indicates that within the laser spot diameter of 3.4 µm, the thickness of the water layer grows to 5 µm, which clearly cannot be assumed to have uniform thickness. Hence, the conventional FDTR model cannot be used.

By increasing the substrate temperature and thus the resulting evaporation rate, the meniscus shape will change. Wang et al. found that increasing the superheat for an evaporating meniscus of octane increased the contact angle.[1] As such, we obtain the ultimate shape of the meniscus from the neural network analysis, as described in the main text.

Table S1. Properties and boundary conditions used in theoretical meniscus shape calculation, evaluated at a temperature of 295 K.

Property Name	Property Value
Water surface tension σ (N/m)	0.072
Hamaker constant A (J)	3.261×10^{-20} (Ref. 2)
Liquid water density ρ (kg/m^3)	997
Acceleration due to gravity g (m/s^2)	9.8
Absorbed water film thickness $t(0)$ (nm)	3.3
Initial slope for a wetting film $t'(0)$	0
Curvature in the far field κ_{far} (m^{-1})	350
Optical light path of the cuvette (mm)	5
Contact angle between water and gold	58° (Ref. 3)
Contact angle between water and cuvette window (SiO_2)	55° (Ref. 4)

S4 Finite element simulation setup

S4.1 Validation

To justify the use of finite element simulations to emulate the FDTR experiment, bulk liquid and bulk vapor setups are first examined. These two setups are chosen because the standard theoretical FDTR model is appropriate when the materials thicknesses are uniform. For the bulk liquid setup, the water thickness is set to be 3.5 µm. This thickness is calculated based on the heat penetration depth d_h, a characteristic length scale describing the depth at which the vapor-liquid interface is not directly affected by the modulated laser. d_h is defined as[5]

$$d_h = \sqrt{\frac{k}{\rho c \pi f}}, \tag{S6}$$

where k is the thermal conductivity of water, ρ is the density of water, c is the heat capacity of water, and f is the pump modulation frequency. For the smallest modulation frequency used in this work (100 KHz), d_h is calculated to be 0.7 µm. The thickness of the water layer is taken to be five times the maximum d_h (3.5 µm) to eliminate the influence of its outer surface.[6] Above this value, the water region is considered bulk because changes in the water thickness cannot be captured in the phase lag as the signal-to-noise ratio decreases. Similarly for the quartz layer, its thickness is calculated to be 8.5 µm to reach a bulk-like response. A 70 nm Au layer and a 200 nm PMMA layer are sandwiched between the water and the quartz to emulate the experimental setup. The geometry is shown in Fig. S5(a). Based on an axisymmetric heat transfer assumption and the Gaussian distribution of the laser intensity, only half of the laser impacting area is modeled. The cross section area ($\delta \times z$) is 30 µm×15 µm. All the boundary conditions are set to be adiabatic. For the bulk vapor setup, the water layer is deleted and the adjacent gold surface is set to be adiabatic.

The finite element simulation phase lags at different pump modulation frequencies are shown in Fig. S5(b) using brown (liquid) and blue (vapor) diamonds. The phase lags calculated from the theoretical FDTR model for the bulk liquid and bulk vapor setups are shown as brown and blue squares. The finite element simulation results agree well with the theoretical FDTR model values for all tested frequencies in both systems with the maximum difference being 0.18 degrees.

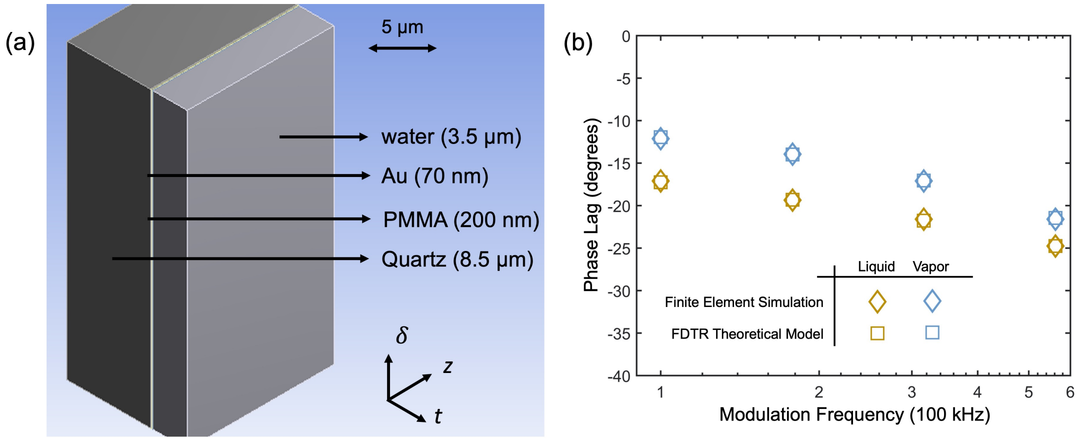

Figure S5. (a) Finite element simulation geometry for the bulk liquid setup. Thickness for each layer is shown in the bracket. For the bulk vapor setup, the water layer is deleted. (b) Comparison between the phase lag calculated from the finite element simulation and that from the theoretical FDTR model. Four different pump modulation frequencies are simulated.

S4.2 Meniscus evaporation

The finite element simulation geometry for the meniscus evaporation is shown in Fig. S6. The heat flux center that models the laser beams is moved along the δ axis to simulate the cases when the lasers are scanned towards the vapor and liquid sides of the meniscus. A summary of the parameter range for all the features is provided in Table S2. 2653 distinct finite element simulation systems with different combinations of the eight features are simulated and used in the neural network training process.

The ranges for the frequency f and laser spot radius r are based on observations in the FDTR experiments. The range for the distance between the laser center (incoming distributed heat flux) and the meniscus edge Δ_{laser} is chosen to ensure that the bulk liquid and bulk vapor regions have been included. The phase lags do not change as the heat flux center moves outside of the chosen range. $\Delta_{\text{laser}} = 0$ is where the meniscus starts. $\Delta_{\text{laser}} < 0$ refers to simulations where the heat flux center is located on the liquid side and $\Delta_{\text{laser}} > 0$ refers to where it is on the vapor side, as shown in Figs. S6(a) and S6(b). The range for the PMMA thermal conductivity is chosen based on literature values.[7,8]

The h_{evap} profile is built from three straight lines, as shown in Fig. S7(a) in red. There are three key features: the peak value $h_{\text{evap,peak}}$, the thickness at the peak t_{peak}, and the thickness at the end, t_{end}. h_{evap} takes on its bulk room temperature value of 0.01 MW/m^2-K after reaching t_{end}.[9–11] An example of an h_{evap} profile in a finite element simulation system is shown in Fig. S7(b). As shown in Table S2, $h_{\text{evap,peak}}$ is

Table S2. Parameter range in finite element simulations generated for the neural network training

Parameter Name	Lower bound	Upper bound
Frequency for the given periodic heat flux f (Hz)	10^5	5×10^6
Spot radius used for the heat flux Gaussian profile r (µm)	1.4	2.1
Δ_{laser} (µm)	-6	3
Thermal conductivity of PMMA k_{PMMA} (W/m-K)	0.175	0.300
$h_{\text{evap,peak}}$ (MW/m^2-K)	0.02	10.00
t_{peak} (nm)	12	100
t_{end} (µm)	0.2	3.5
Interface shape coefficient c (µm^{-1})	0.2	10.0

varied between 0.02 and 10 MW/m^2-K, t_{peak} is varied between 12 and 100 nm, and t_{end} is varied between 0.2 and 3.5 µm. The lower bound for $h_{\text{evap,peak}}$ is chosen based on the bulk value and the upper bound is based on literature values.[12,13] The lower bound for t_{peak} is based on the starting thickness of the meniscus (10 nm) in the finite element simulation due to the meshing resolution and the upper bound is a large value that no best fit is able to reach. For t_{end}, the lower bound is chosen to be larger than the upper bound for t_{peak} and the upper bound is the bulk thickness used for water in the finite element simulation.

The water meniscus is created in the form of a parabola with c being the interface shape coefficient. The meniscus thickness starts at 10 nm for meshing purposes and stops at a thickness of 3.5 µm based on the heat penetration depth calculation from Eq. (S6). The c values used in the finite element simulations to generate the neural network training data are between 0.2 and 10.0 µm^{-1}. The lower c bound is taken to be half of the value for the theoretically calculated shape for a non-evaporating meniscus and the upper bound is a large value that no best fit is able to reach.

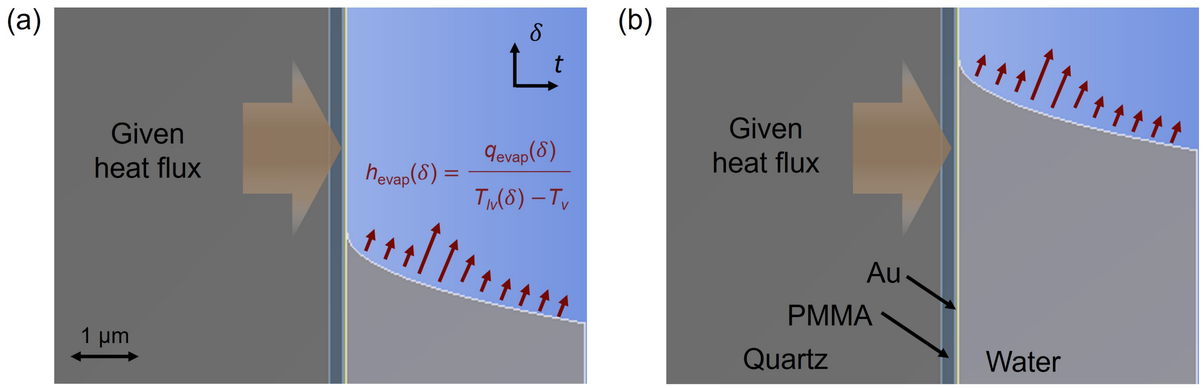

Figure S6. Finite element simulation geometry for the meniscus evaporation when the lasers are moved to (a) vapor region, and (b) liquid region.

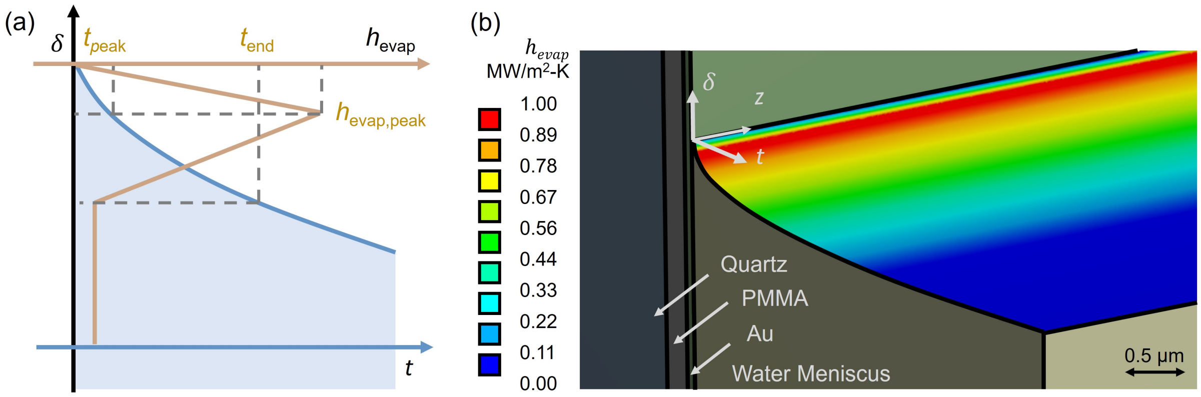

Figure S7. (a) Schematic drawing of the defined h_{evap} profile, with respect to the thickness profile, δ. Red lines represent the h_{evap} profile and the blue curve represent the water thickness profile. (b) h_{evap} profile in the finite element simulation as a convection boundary condition. This h_{evap} profile has a peak value of 1 MW/m^2-K at a film thickness of 30 nm and ends where the film thickness is 2 µm.

S5 Neural network

Scikit-Learn packages are used for the neural network structure selection and training.[14] The finite element simulation data are normalized before feeding into the neural network. The neural network structure is selected through a combination of random search to target the optimal range and grid search to fine tune the hyperparameters. Six hyperparameters are tested during the random search: activation function, hidden layer size, optimization solver, learning rate, max iteration, and tolerance. A five-fold cross validation is used to select the neural network structure with the highest score. The final neural network structure is a three-layer neural network. The two hidden layers have 12 and 15 nodes. The logistic activation function is chosen and the Limited-memory Broyden–Fletcher–Goldfarb–Shanno (L-BFGS) algorithm is used as the optimization solver. The max iteration is set to 10000 and the tolerance is set to 0.001. A mean cross validation accuracy of 0.997 is achieved with the chosen neural network structure.

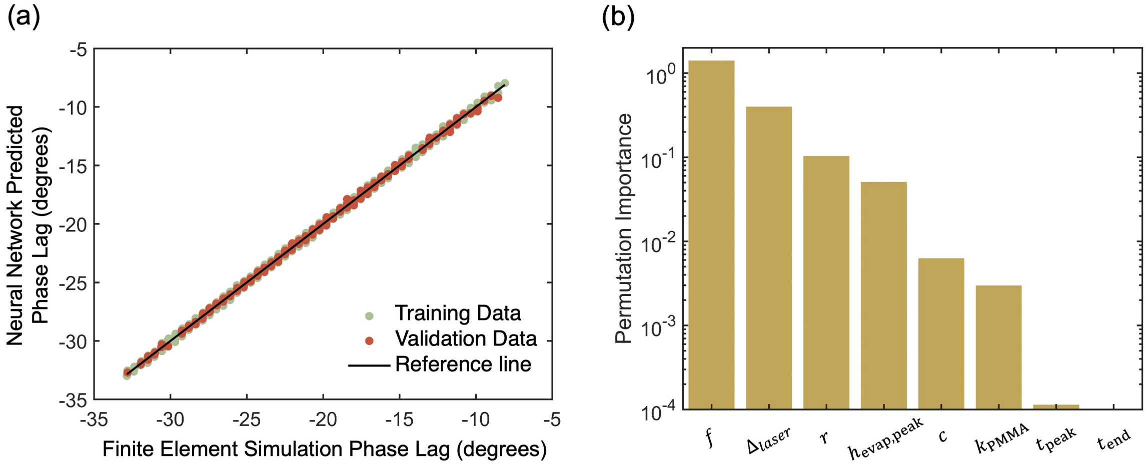

Figure S8. (a) Parity plot to show the performance of the trained neural network. (b) Permutation importance of the features.

2653 finite element simulations were run. Ten percent of the data are randomly selected for validation purpose. The parity plot in Fig. S8(a) shows the goodness of the constructed neural network with random seed values being zero. The horizontal axis is the phase lag provided by the finite element simulations and the vertical axis is the phase lag predicted by the trained neural network. As can be seen from Fig. S8(a), the predicted training and validation data all locate close to the reference line, meaning that the predictions agree well with the provided values. The correlation coefficients for the training and validation data are

both above 0.99.

The permutation importance values of the input features are listed in Fig. S8(b) with a log scale comparison. The most sensitive input feature is the laser modulation frequency. This result is advantageous because the modulation frequency can be accurately extracted from the FDTR experiment. The least sensitive parameters are t_{peak} and t_{end}, which means that these two features do not significantly affect the phase lag compared to the other parameters, such as $h_{\mathrm{evap,peak}}$ and c.

S6 Powell optimization

To extract the feature values when the trained neural network is used to fit the experimental data, the Powell optimization technique is used. Bounds are set for the six features during the Powell optimization, as shown in Table S3.

One additional constraint is imposed on the laser position relative to the meniscus edge, Δ_{laser}. Since the data taken from FDTR have a 1 μm interval, the laser position obtained from the optimizations between different data points need to be consistent with the operating condition. For each FDTR scan, the first data point in the bulk liquid is taken to be the starting point of Δ_{laser}, denoted by $\Delta_{\text{laser,0}}$. The data taken next to this position should always have a position of $\Delta_{\text{laser,0}} + 1\,\mu\text{m}$, and subsequently, the other points will be at integer intervals of $1\,\mu\text{m}$ away from $\Delta_{\text{laser,0}}$.

Table S3. Feature ranges during Powell optimization

Parameter Name	Lower bound	Upper bound
Spot radius of the lasers r (μm)	1.4	2.1
Initial laser position $\Delta_{\text{laser,0}}$ (μm)	-9	-5
$h_{\text{evap,peak}}$ (MW/m^2-K)	0.1	10.0
t_{peak} (nm)	12	100
t_{end} (μm)	0.2	3.4
interface shape coefficient c (μm^{-1})	0.2	10.0

S7 Histograms for $h_{\text{evap,peak}}$ and interface shape coefficient c

To include the randomness in the prediction caused by random seeds when training the neural network, one thousand neural networks with different seeds were created. Hence, one thousand sets of extracted features are obtained for each FDTR experiment. Histograms of the interface shape coefficient c and $h_{\text{evap,peak}}$ for the six FDTR experiments are shown in Fig. S9, where each histogram contains one thousand values. The final histograms for c and $h_{\text{evap,peak}}$ shown in Figs. 3(a) and 3(b) are the combinations of Figs. S9(a)-S9(f) and S9(g)-S9(l).

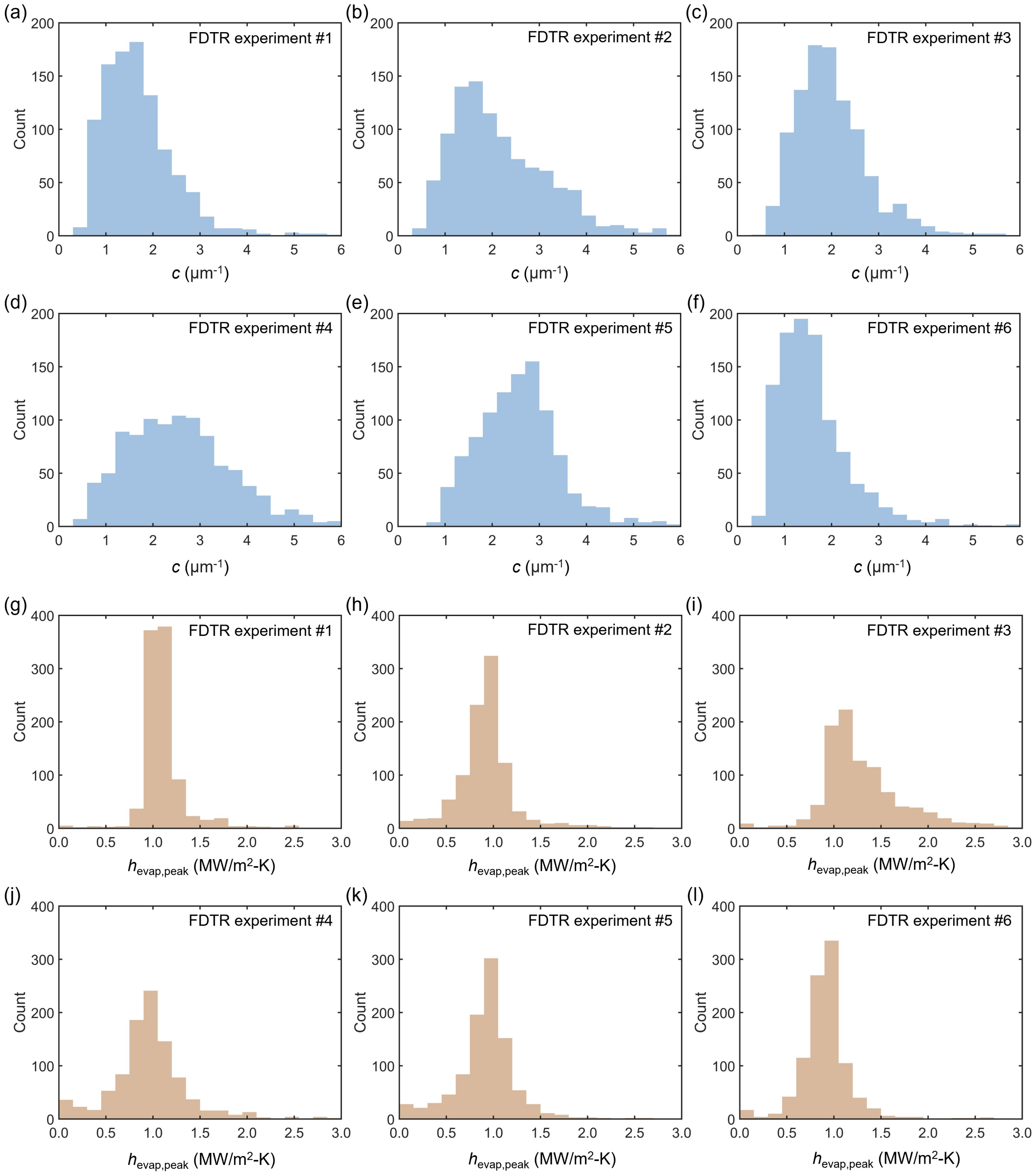

Figure S9. Histograms for (a)-(f) interface shape coefficient c and (g)-(l) $h_{\mathrm{evap,peak}}$ of the six FDTR experiments.

S8 Two-sample Kolmogorov-Smirnov null hypothesis test

The two-sample Kolmogorov-Smirnov null hypothesis test is performed to check the similarity between the histograms in Fig. S9. 25 values are randomly drawn from each of two histograms. The sample size of 25 is calculated using Altman's monogram with a standardized difference of 0.8, significance of 5%, and test power of 80%.[15] To eliminate the impact of randomness in the drawn data, the process of repeated 100 times for each pair of histograms. A pair is accepted as being from the same population if it passes the test in more than half of the 100 times. With six data sets, a total of 15 pairs are tested for each of $h_{\text{evap,peak}}$ and c. Combining the test results on $h_{\text{evap,peak}}$ and c: FDTR experiments #1 and #3 are sampled from one population, FDTR experiments #2, #4, and #5 are from a second population, and FDTR experiments #2 and #6 are from a third population. The results do not prove that all the distributions are drawn from the same population. To illustrate the data spread, the six histograms are combined to obtain the distributions for c and $h_{\text{evap,peak}}$ plotted in Figs. 3(a) and 3(b).

S9 Evaporation mass flux calculations

The Hertz-Kundsen-Schrage (HKS) relation for the evaporative mass flux is

$$\dot{m}''_{\text{evap,HKS}} = \frac{2\times\alpha(T_{lv})}{2-\alpha(T_{lv})}\sqrt{\frac{M}{2\pi R}}\left[\frac{P_{eq}(T_{lv})}{\sqrt{T_{lv}}} - \frac{P_v}{\sqrt{T_v}}\right]. \tag{S7}$$

Here, M is the molar mass of the liquid, R is the universal gas constant, T_{lv} is the liquid-vapor temperature, T_v is the vapor temperature, the pressure P_v is calculated as the saturation vapor pressure at T_v, α is the temperature-dependent mass accommodation coefficient extracted from molecular dynamics simulations,[16] and P_{eq} is the equilibrium pressure,[17]

$$P_{eq}(T_{lv}) = P_{sat}(T_{lv})\times\ \exp\left[\frac{P_{eq}(T_{lv})-P_{sat}(T_{lv})-(P_d(t)+P_c)}{\rho_l T_{lv} R/M}\right]. \tag{S8}$$

Here, $P_{sat}(T_{lv})$ is the temperature-dependent saturation vapor pressure,[18] t is the film thickness, ρ_l is the liquid density, P_d is the disjoining pressure, and P_c is the capillary pressure. P_d and P_c are calculated using Eqs. (S3) and (S2). Since the meniscus shape is approximated as a parabola, the calculated P_c is an approximation.

To compare our experimental results with the HKS mass flux prediction, we calculate the mass flux using the mass-energy balance.

$$\dot{m}''_{\text{evap,direct}} = \frac{h_{\text{evap}}(T_{lv})\times(T_{lv}-T_v)}{h_{fg}}, \tag{S9}$$

where h_{fg} is the temperature-dependent latent heat of vaporization.[19]

The position-dependent liquid-vapor temperature T_{lv} required in Eqs. (S7) and (S9) is extracted from finite element simulations run with only the direct current (DC) component of the heat flux applied. The median values of h_{evap} and c from the fits to the neural networks are used in these simulations. The heat flux is centered in the middle of the meniscus width (position = 0.7 µm) because the FDTR signal is most sensitive to this middle position during the optimization process. At the liquid-vapor interface, the h_{evap} profile is set as the obtained profile with $h_{\text{evap,peak}} = 1.0$ MW/m^2-K, $t_{\text{peak}} = 15$ nm, and $t_{\text{end}} = 1.6$ µm.

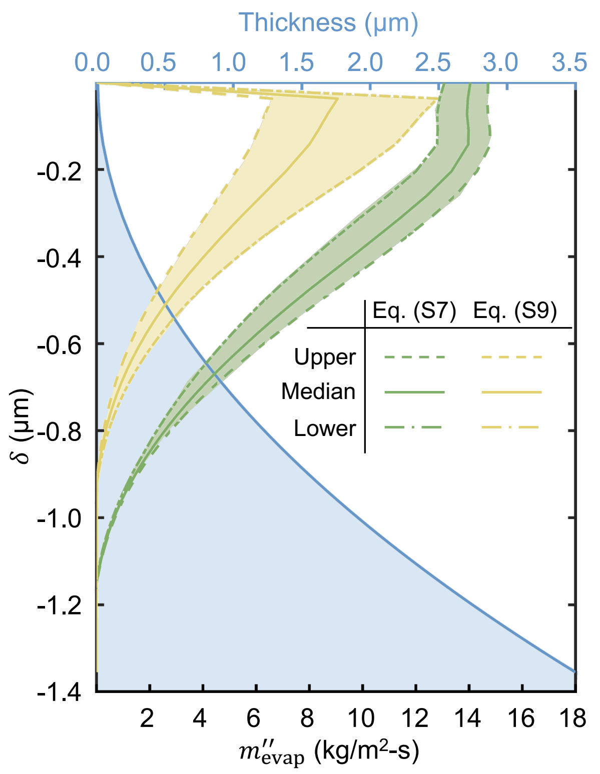

Figure S10. Comparison between the mass flux calculated using Eq. (S7) and that calculated using Eq. (S9). The dashed curve and dash-dotted curve are plotted by keeping the median value of the interface shape coefficient c as 1.9 μm^{-1} and varying $h_{\mathrm{evap,peak}}$ to its upper and lower bound.

The mass fluxes calculated using Eqs. (S7) and (S9) are plotted in Fig. S10. The meniscus shape is obtained using the median value of c, which is 1.9 μm^{-1}. The solid curves are calculated using the median value of $h_{\mathrm{evap,peak}}$ (1.0 MW/m^2-K). The dashed curve and dash-dotted curve are calculated based on the lower and upper bound of $h_{\mathrm{evap,peak}}$. As the $h_{\mathrm{evap,peak}}$ varies, the finite element simulation needs to be performed again to obtain a new T_{lv} profile.

Because the meniscus thickness starts at 10 nm in the finite element simulation due to limitations on the meshing resolution, P_d does not influence the calculated $\dot{m}''_{\mathrm{evap,HKS}}$. The two peak mass fluxes differ by about 40%. We hypothesize the difference at the peak to be to be caused by the mass accommodation coefficient α estimation[20] and the non-zero averaged vapor molecule velocity tangential to the interface.[16] In terms of the difference in the two $\dot{m}''_{\mathrm{evap}}$ profile shapes, we hypothesise the causes to be: (i) The assumed h_{evap} is compromised of linear segments that may not capture all the features of the true profile; (ii) The trained neural network is least sensitive to t_{end}.

References

1. Wang, H., Garimella, S. V. & Murthy, J. Y. Characteristics of an evaporating thin film in a microchannel. *international journal heat mass transfer* **50**, 3933–3942 (2007).

2. Narayanan, S., Fedorov, A. G. & Joshi, Y. K. Interfacial transport of evaporating water confined in nanopores. *Langmuir* **27**, 10666–10676 (2011).

3. Cognard, J. Adhesion to gold: A review. *Gold Bull.* **17**, 131–139 (1984).

4. Mohsin, K. *et al.* Development of self-nanoemulsifying drug delivery systems for the enhancement of solubility and oral bioavailability of fenofibrate, a poorly water-soluble drug. *Int. journal nanomedicine* **11**, 2829 (2016).

5. D'Esposito, R., Frégonèse, S. & Zimmer, T. Electrothermal characterization, tcad simulations, and physical modeling of advanced sige hbts. In *Nanoelectronics*, 27–94 (Elsevier, 2019).

6. Cahill, D. G. & Pohl, R. O. Thermal conductivity of amorphous solids above the plateau. *Phys. review B* **35**, 4067 (1987).

7. Yamaguchi, S. *et al.* Anisotropic thermal conductivity measurement of organic thin film with bidirectional 3 ω method. *Rev. Sci. Instruments* **92**, 034902 (2021).

8. Katz, J. S. *et al.* Nanostencil fabrication with double exposure optical lithography for scalable resist-free patterning of metal on polymers. In *2018 Transducer Research Foundation Hilton Head Workshop* (2018).

9. Lemmon, E., Huber, M. & McLinden, M. Nist standard reference database 23: Reference fluid thermodynamic and transport properties-refprop, version 8.0 (2007).

10. Nishikawara, M. & Nagano, H. Optimization of wick shape in a loop heat pipe for high heat transfer. *Int. J. Heat Mass Transf.* **104**, 1083–1089 (2017).

11. Hodes, M., Steigerwalt Lam, L., Cowley, A., Enright, R. & MacLachlan, S. Effect of evaporation and condensation at menisci on apparent thermal slip. *J. Heat Transf.* **137** (2015).

12. Zhao, J.-J., Duan, Y.-Y., Wang, X.-D. & Wang, B.-X. Effects of superheat and temperature-dependent thermophysical properties on evaporating thin liquid films in microchannels. *Int. J. Heat Mass Transf.* **54**, 1259–1267 (2011).

13. Lay, J. H. & Dhir, V. K. Shape of a Vapor Stem During Nucleate Boiling of Saturated Liquids. *J. Heat Transf.* **117**, 394–401 (1995).

14. Pedregosa, F. *et al.* Scikit-learn: Machine learning in Python. *J. Mach. Learn. Res.* **12**, 2825–2830 (2011).

15. Cohen, J. *Statistical power analysis for the behavioral sciences* (Academic press, 2013).

16. Chandra, A. & Keblinski, P. Investigating the validity of schrage relationships for water using molecular dynamics simulations. *The J. Chem. Phys.* **153**, 124505 (2020).

17. Faghri, A. *Heat pipe science and technology* (Global Digital Press, 1995).

18. Lide, D. R. *CRC handbook of chemistry and physics*, vol. 85 (CRC press, 2004).

19. Pellicer, J., García-Morales, V., Guanter, L., Hernández, M. & Dolz, M. On the experimental values of the water surface tension used in some textbooks. *Am. J. Phys.* **70**, 705–709 (2002).

20. Persad, A. H. & Ward, C. A. Expressions for the evaporation and condensation coefficients in the hertz-knudsen relation. *Chem. reviews* **116**, 7727–7767 (2016).
